# Association between Serum Vitamin C and the Blood Pressure: A Systematic Review and Meta-Analysis of Observational Studies

**DOI:** 10.1155/2020/4940673

**Published:** 2020-04-29

**Authors:** Li Ran, Wenli Zhao, Xiaodong Tan, Hongwu Wang, Kaito Mizuno, Ken Takagi, Ye Zhao, Huaien Bu

**Affiliations:** ^1^Department of Occupational and Environmental Health, School of Health Sciences, Wuhan University, Wuhan 430071, China; ^2^Department of Acupuncture and Moxibustion, Suzuka University of Medical Science, Suzuka 510-0293, Japan; ^3^College of Traditional Chinese Medicine, Tianjin University of Traditional Chinese Medicine, Tianjin 300193, China; ^4^Faculty of Health Science, Suzuka University of Medical Science, Suzuka 510-0293, Japan; ^5^Institute of Traditional Chinese Medicine, Suzuka University of Medical Science, 1001-1 Kishioka, Suzuka 510-0293, Japan; ^6^Qingdao Academy of Traditional Chinese Medicine, Shandong University of Traditional Chinese Medicine, Qingdao 266112, China

## Abstract

**Background:**

Hypertension is regarded as a major and independent risk factor of cardiovascular diseases, and numerous studies observed an inverse correlation between vitamin C intake and blood pressure.

**Aim:**

Our aim is to investigate the relationship between serum vitamin C and blood pressure, including the concentration differences and the correlation strength.

**Method:**

Two independent researchers searched and screened articles from the National Library of Medicine, Cochrane Library, Web of Science, China National Knowledge Infrastructure, VIP databases, and WANFANG databases. A total of 18 eligible studies were analyzed in the Reviewer Manager 5.3 software, including 14 English articles and 4 Chinese articles.

**Results:**

In the evaluation of serum vitamin C levels, the concentration in hypertensive subjects is 15.13 *μ*mol/L lower than the normotensive ones (mean difference = −15.13, 95% CI [-24.19, -6.06], and *P* = 0.001). Serum vitamin C has a significant inverse relation with both systolic blood pressure (Fisher′s *Z* = −0.17, 95% CI [-0.20, -0.15], *P* < 0.00001) and diastolic blood pressure (Fisher′s *Z* = −0.15, 95% CI [-0.20, -0.10], *P* < 0.00001).

**Conclusions:**

People with hypertension have a relatively low serum vitamin C, and vitamin C is inversely associated with both systolic blood pressure and diastolic blood pressure.

## 1. Introduction

Cardiovascular diseases (CVDs) are a series of disorders of blood vessels and the heart, primarily including coronary heart disease, cerebrovascular disease, and rheumatic heart disease [1]. Owing to its increasingly high worldwide morbidity and gradual tendency in younger people, CVD is considered to be one of the most serious diseases that damaged public health in the 21^st^ century [2]. The number of deaths due to CVD is increasing year by year. For example, a total of 17.9 million individuals died from cardiovascular events in 2015, which is much higher than that in 1990 [3]. In developing countries, it is reported that more than 75% of CVD deaths occur; approximately 41% of total deaths in China are related to CVD with an annual death toll reaching 3.5 million [4–6].

Individuals with a risk of CVD might manifest with a raised blood pressure (BP) [7]. As researches continuing, numerous epidemiological studies have repeatedly recognized that hypertension is a major and independent risk factor of CVD [1, 8, 9]. As the most common chronic noninfectious disease, hypertension is closely related to several risk factors, including genetics [10, 11], family history [12–14], overweight and obesity [15], tobacco smoking [15–18], physical inactivity [19, 20], and unhealthy dietary intake [17, 20]. Nutrient intake and electrolyte level are complex and varied, but population-based evidence has shown that the consumption of magnesium, sodium, potassium, and calcium is inversely associated with BP [21, 22]. Other studies also revealed the relationship between vitamin C and BP, one of which was that hypertensive patients present a lower intake and serum vitamin C [23]. A variety of observational and interventional studies has additionally reported that vitamin C intake and its concentration status were significantly related to a reduction of resting BP [24]. For example, Kamran et al. [25] found that the correlation between vitamin C intake and systolic BP was -0.02 in uncontrolled hypertensive patients. Yet another study by Yoshioka et al. [26] discovered that serum vitamin C had an inverse and the strongest association with systolic BP. Given the potential oxidation resistance of vitamin C, researchers attributed this association to that vitamin C could prevent the formation of free radicals, thereby reducing the vascular oxidative in the progress of hypertension [27]. However, these preliminary findings have not been confirmed, since some researchers drew an opposite conclusion, like the study conducted by Duthie et al. [28]. Even relevant systematical review and meta-analysis articles have not been found regarding the relationship between them.

To consider the controversial role of vitamin C in the prevention and management of hypertension, we are inspired to conduct this meta-analysis, with a purpose to compare serum vitamin C levels between hypertensive and normotensive individuals. Furthermore, we aim to confirm whether there was a correlation between serum vitamin C and BP and calculate the strength of the relationship.

## 2. Methods

### 2.1. Search Strategy

Two independent reviewers comprehensively searched the National Library of Medicine (PubMed), Cochrane Library, Web of Science (WOS), China National Knowledge Infrastructure (CNKI), VIP databases, and WANFANG databases to obtain relevant studies from their earliest publication up to January 2019. We used the following searching strategy: (Vitamin C OR ascorbic acid OR risk factor) AND (hypertension OR high blood pressure OR blood pressure). There was no limitation to language. All the records were screened and scrutinized independently by two partners. All the studies were screened and selected according to the guidelines for Systematic Reviews of Observational Studies (MOOSE) [29].

### 2.2. Inclusion and Exclusion Criteria

The eligible studies should fulfill the inclusion criteria as follows: (1) investigated the relation between serum vitamin C and blood pressure among hypertensive subjects or normotensives; (2) participants were males or females over 18 years old; (3) observational articles including cross-sectional studies, case-control studies, and cohort studies; (4) provided with Pearson's correlation coefficient (*r*), Spearman's correlation coefficient (*r*_s_), or regression coefficient (*b*); (5) the means and standard deviations (SD) of serum vitamin C were available in case-control studies; and (6) if there were over two similar articles published based on the same sample, the one with higher quality would be included.

Studies were excluded with the following criteria: (1) duplicated or similar articles; (2) nonobservational studies, such as animal testing and intervention experiment; (3) correlation coefficient, means, or SD could not be acquired to calculate the pooled effect size; (4) the level of vitamin C was measured from urine; and (5) participants used supplements with vitamin C beyond the recommended dietary allowances.

### 2.3. Quality Assessments

Considering that different types of observational studies were included in our meta-analysis, we used two methodological quality checklists. The first one was the Newcastle-Ottawa Scale (NOS) [30], assessing case-control and cohort studies. The assessment of NOS was performed on the items of the selection of study population, comparability between cases and noncases, exposure, and the outcome, with a maximum score of 9. We regarded the article awarded a score ≥5 as a high-quality assessment, owing to that the standard validated criteria have not been established [31]. Cross-sectional studies were assessed using 11 items recommended by the Agency for Healthcare Research and Quality (AHRQ) [32]. These items would be answered with “yes,” “unclear,” and “no,” separately scored with 1 and 0.

### 2.4. Data Extraction

We extracted the following data to describe the main characters of each study, including the first author's name, publication year, country conducted in, the age of subjects, sample size, BP value, study design, outcomes (correlation coefficient or means and standard), and variables adjusted for.

### 2.5. Data Conversion

If the Pearson correlation coefficient (*r*) was not provided, the Spearman coefficient (*r*_s_) and regression coefficient (*b*) with standard error (SE) could be used to estimate the *r* value [33]. Details are as follows:

1. *r* was calculated if *r*_s_ was available:

(1) $$\begin{array}{c} r=2\times \sin\left(r_{\text{s}}\times \frac{\pi }{6}\right). \end{array}$$

(2) *r* was calculated if *b* was available:

“SE_*b*_” is the standard error of *b*, so

(2) $$\begin{array}{c} t=\frac{b}{{\text{SE}}_{b}}.\\ r=\frac{t}{\sqrt{\left(t^{2}+n-2\right)}} \end{array}$$

We performed Fisher's *Z* transformation to convert every correlation coefficient to an approximately normal distribution, and then the pooled effect size was weighted with the inverse variance. Fisher's *Z* transformation was according to the following formulates [34]:

(3) $$\begin{array}{c} {\text{Fisher}}^{\prime }\text{s }Z=0.5\times \ln\frac{1+r}{1-r},\\ V_{z}=\frac{1}{n-3},\\ S_{E}=\sqrt{V_{z}}.\\ \text{Summary }r=\frac{e^{2z}-1}{e^{2z}+1}. \end{array}$$

where *Z* stands for the value of summary Fisher's *Z*.

### 2.6. Statistical Analysis

The mean difference (MD) was applied for continuous variables in case-control studies (or cross-sectional studies) while Fisher's *Z* value was applied for correlation coefficients to calculate the pooled effect size; for both, the corresponding 95% confidence intervals (CI) were available and the forest plots were used to display the results graphically. Subgroup analyses were performed by sex, with or without hypertension, antihypertensive drugs, level of vitamins A and E, study area, and sample size. All the statistical analysis was conducted with the Reviewer Manager (RevMan) 5.3 software.

Heterogeneity was detected by Q-statistics, derived from the Chi-squared test and I-squared (inconsistency). Notable heterogeneity was indicated when the *P* value was below 0.05 or an *I*^2^ value was above 50%, and in this case, a random-effects model was preferred. Then, a sensitivity analysis would be performed to investigate the potential sources of heterogeneity. Publication bias was visually assessed with the funnel plot, and Begg's test and Egger's test could also be applied with the Stata 14.0 software when necessary.

## 3. Results

### 3.1. Study Selection

**Figure 1:**
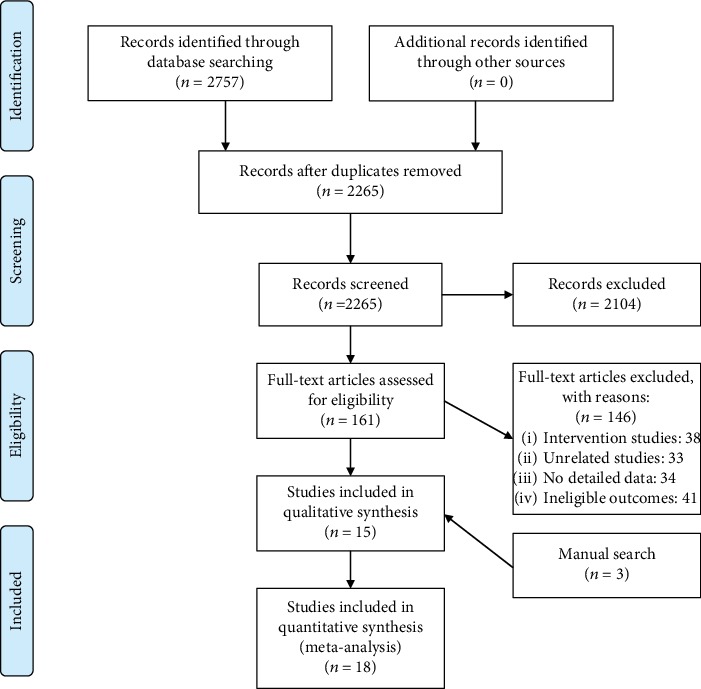
PRISMA 2009 flow diagram.

A total of 2757 articles were searched from English and Chinese databases. There were 2104 articles excluded by screening the titles and abstracts, and finally, 18 eligible articles [35–52] were included in our meta-analysis based on full-text review and manual search. The study selection procedure is outlined in Figure 1

### 3.2. Study Characteristics and Quality

As shown in Table 1, the selected articles included 11 cross-sectional studies and 7 case-control studies. These studies comprised 22200 observational subjects and were conducted from the year 1990 to 2017. Of the 18 articles, 14 were published in the English language, and 4 were in Chinese.

Assessed with NOS, all the case-control studies yield a high quality averaging with 7.143 scores. And the result of AHRQ indicates a moderate quality with all cross-sectional studies scoring between 4 and 7.

### 3.3. Meta-Analysis of Outcome

#### 3.3.1. Serum Vitamin C Concentration

**Figure 2:**
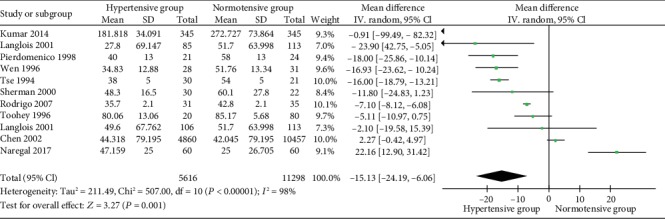
Forest plot of the meta-analysis of serum vitamin C concentration in hypertensive and normotensive subjects.

The level of serum vitamin C between hypertensive subjects and normotensives is described in Figure 2, which involved 10 studies composing of 16914 participants [37, 40, 43–46, 50]. Owing to high heterogeneity (Cochrane Q test = 507.00, degrees of freedom (df) = 10, *P* < 0.00001, *I*^2^ = 98%), the analysis was conducted on the random-effects model. It was obvious that the serum level of vitamin C of hypertensive subjects was 15.13 *μ*mol/L lower than the normotensives (MD = −15.13, 95% CI [-24.19, -6.06], *P* = 0.001).

Due to the high heterogeneity, a sensitivity analysis was conducted, in which one single study was omitted at a time while the others were recalculated to estimate if the result could affect markedly. We found that the average level of vitamin C concentration in Kumar's study [50] was several times higher than the others, which might indicate to some mistakes in raw data. It was additionally found that vitamin C intake between the groups was significantly different. The *I*^2^ value reduced from 98% to 94% after removing this study, whereas it remained stable after omitting other studies. A subgroup analysis was subsequently performed, revealing that hypertensive subjects who took antihypertensive drugs had a 15.97 *μ*mol/L lower serum vitamin C compared with normotensive ones. And no obvious heterogeneity was found.

#### 3.3.2. The Correlation between Vitamin C and Blood Pressure

**Figure 3:**
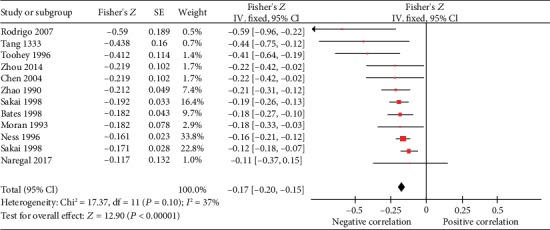


*(1) Systolic Blood Pressure*. The correlation between serum vitamin C and the systolic blood pressure (SBP) was described in 12 studies, 1 of which was excluded in our meta-analysis for the missing value of SE [47]. As illustrated in Figure 3 the analysis was conducted with fixed-effects model due to a low heterogeneity (Cochrane Q test = 17.37, df = 11, *P* = 0.10, *I*^2^ = 37%). The pooled Fisher's *Z* was -0.17 (Fisher′s *Z* = −0.17, 95% CI [-0.20, -0.15], *P* < 0.00001), indicating a reverse relation between serum vitamin C concentration and SBP significantly. And the summary *r* value was -0.168 calculated with the formula above.

**Figure 4:**
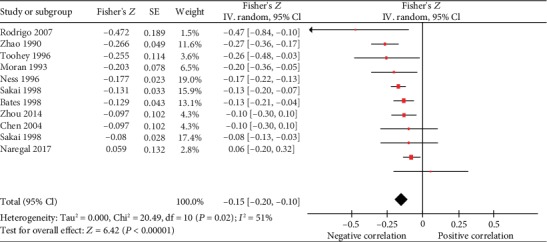


*(2) Diastolic Blood Pressure*. By conducting on a random-effects model, serum vitamin C concentration was inversely correlated to diastolic blood pressure (DBP) with Fisher's *Z* value of -0.15 (Fisher′s *Z* = −0.15, 95% CI [-0.20, -0.10], *P* < 0.00001). The summary *r* was -0.149, and there was a moderate heterogeneity (Cochrane Q test = 20.49, df = 10, *P* = 0.02, *I*^2^ = 51%) (Figure 4

*(3) Subgroup Analysis*. Reflected in Table 2, the subgroup analyses of the association between plasma vitamin C and blood pressure were carried out based on gender, with or without hypertension, antihypertensive drugs, level of vitamins A and E, study areas, and the sample size. Results of all subgroups revealed that serum vitamin C was negatively correlated to SBP and DBP, with significance. In the analysis of SBP, the heterogeneity in each subgroup was not quite high, except for male subjects (Cochrane Q test = 12.39, df = 5, *P* = 0.03, *I*^2^ = 60%) and hypertensive subjects (Cochrane Q test = 7.00, df = 1, *P* = 0.008, *I*^2^ = 86%). In the analysis of DBP, there was an obvious heterogeneity in male (Cochrane Q test = 14.24, df = 5, *P* = 0.01, *I*^2^ = 65%), female (Cochrane Q test = 3.76, df = 1, *P* = 0.05, *I*^2^ = 73%), and studies in Asia area (Cochrane Q test = 12.96, df = 5, *P* = 0.02, *I*^2^ = 61%).

*(4) Adjustment of Main Confounders*. Two studies [38, 41] provided correlation coefficients adjusting for potential factors. After adjustment for the confounders of age, sex, and body mass index (BMI), the association remained the same. As displayed in Table 2, there was a significant negative correlation between serum vitamin C and SBP with low or moderate heterogeneity, except for male adjusted with age or age and BMI. The association between DBP and plasma vitamin C were inverse and stable after adjustment for confounders.

### 3.4. Publication Bias

**Figure 5:**
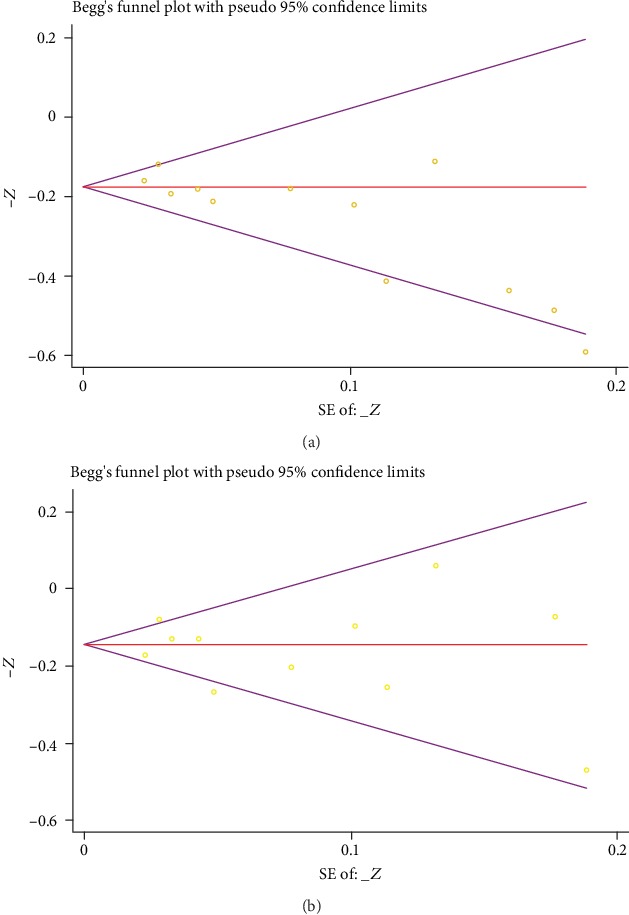


The funnel plot in the comparison of plasma vitamin C and SBP is suggestive of publication bias, and thus, we conducted Begg's test and Egger's test after that. Summarized in Figure 5(a), the results of Begg's test (*P* = 0.015) and Egger's test (*P* = 0.003) showed notable evidence of publication bias. But in the comparison of plasma vitamin C and DBP, the results of Begg's test (*P* = 0.819) and Egger's test (*P* = 0.725), as well as a funnel plot, manifested no distinctive publication bias (Figure 5(b)).

## 4. Discussion

Previous studies have observed an elevation of plasma marker of oxidative stress in the elderly hypertensive subjects, suggesting that oxidative stress may be the mechanism of hypertension. For this reason, antioxidants prevent free radicals from oxidizing or reduce free radical formation, thus, protecting cell membrane pumps from oxidative damage, which might be the reason and evidence for using it in treating hypertension. As a result, vitamin C, the most effective water-soluble antioxidant in human plasma, is regarded to have a protective role against hypertension disease and CVDs [53].

On the bases of free radical theory, researchers demonstrated that ascorbic-free radicals are first formed and then converted to dehydroascorbic acids and semidehydroascorbic acids, scavenging highly reactive free radicals and oxides, including superoxide anions (O_2−_), hydroxyl radicals (OH·), organic free radicals (R·), and peroxy radical (ROO·). Therefore, vitamin C may bring vascular endothelial cells injured by oxidization into a reduced state to recover their functionality and keep the vessels pliable [54, 55]. Apart from this, the oxidation resistance of vitamin C may also manifest itself in the facilitation of glutathione (GSH) synthesis, while both the reduction of GSH and the decreased activity of glutathione peroxidase (GSH-Px) would probably trigger hypertension [56, 57]. Currently, studies observed a relatively lower concentration in hypertensive subjects, and the results of our meta-analysis confirmed it. Just as our results exhibited, the mean serum vitamin C level of hypertension was 15.13 *μ*mol/L lower compared to nonhypertensions. Additionally, both the hypertensive and the normotensive subjects have a significant inverse correlation to SBP and DBP.

Even though vitamin C has an antihypertensive effect by decreasing oxidative stress and improving vascular endothelial function, there is still no validated conclusion from it. Yet, several limitations and weaknesses in our research findings were aroused, which would be a research field or main concerns in the future.

First of all, there is an obvious individual difference. This difference is embodied in two aspects: serum vitamin C concentration and correlation coefficient. To be specific, hypertensives' serum vitamin C ranges from 27.800 ± 69.147 *μ*mol/L to 181.818 ± 34.091 *μ*mol/L, while normotensives' ranges from 25.000 ± 26.705 *μ*mol/L to 272.727 ± 73.864 *μ*mol/L. Besides, the *r* value of SBP is from -0.53 to -0.016, while DBP's *r* value is between -0.269 and 0.059. We speculated this difference may come from the age or the individual differences in population; therefore, subgroup analysis of age and race should be conducted if provided in original studies.

In the next place, causal associations between plasma vitamin C and BP cannot be inferred, because all the studies included in our meta-analysis are case-control and cross-sectional studies. What is more, the association is not very strong. The summary *r* value between serum vitamin C and DBP was -0.149. In terms of our included literature, serum vitamin C presents a weak correlation to SBP with a comprehensive *r* value of -0.168, whereas DBP showed a correlation of -0.149. And it is notable that the correlation to SBP in hypertensive ones is insignificant (Fisher′s *Z* = −0.16, 95% CI [-0.36, 0.03], *P* = 0.10).

Last, due to the heterogeneity and publication bias existing in our meta-analysis, we should be more cautious before jumping to any conclusion. In the evaluation of serum vitamin C, despite a lower concentration was identified in hypertensive subjects, there is a high heterogeneity. Through the subgroup analysis, all hypertensive subjects sticking with antihypertensive drugs consistently showed much lower serum vitamin C (15.97 *μ*mol/L), whereas those who did not take drugs showed high heterogeneity. We speculated that antihypertensive drugs might consume serum vitamin C. It was additionally found that the serum level of vitamins A and E did not cause the heterogeneity mainly, and it was similar in the correlation analysis. Hence, after comprehensively considering the heterogeneity and publication bias, the results are more stable in females, nonhypertensives, or hypertensives taking antihypertensive drugs, but it calls for more original studies to verify.

For all mentioned above, more details should be considered in further studies. On one hand, prospective studies with high qualities are required to link vitamin C deficiency to the risk of HBP; on the other, a meta-analysis should be conducted on the relation between vitamin C intake and hypertension.

## 5. Conclusion

Hypertensives are exposed in a lower serum vitamin C concentration. Serum vitamin C generally shows a negative relation to SBP and DBP.

## Figures and Tables

**Table 1:** The characters and the quality assessment of included studies.

First author	Year	Study area	Sample size			Age	BP value	Study design	Outcome	Adjusted variable	Quality score
			Male	Female	Overall		(SBP/DBP)				
Zhao	1990	China	NA	NA	416	40-59	NA	Cross-sectional	Correlation coefficient	/	5
Moran	1993	USA	60	108	168	19-70	111.6 ± 0.9/72.3 ± 0.7	Cross-sectional	Correlation coefficient	/	6
Tse	1994	UK	39	34	73	C: 59.6 ± 11.7 N: 51.8 ± 12.3	C: 181 ± 27/98 ± 13 N: 125 ± 15/76 ± 9	Case-control	M ± SD Regression coefficient	Age, sex, smoking	7
Wen	1996	Ireland	32	27	59	C: 58.3 ± 19.5 N: 50.6 ± 12.7	NA	Case-control	M ± SD	/	7
Toohey	1996	USA	42	126	168	F: 49.0 ± 1.4 M: 44.5 ± 2.1	F: 118.8 ± 1.7/77.2 ± 0.9 M: 121.0 ± 3.3/79.0 ± 1.7	Cross-sectional	Correlation coefficient	/	4
Ness	1996	UK	835	1025	1860	45-75	135.8 ± 18.5/82.5 ± 11.3	Cross-sectional	Correlation coefficient Regression coefficient	Age, sex and BMI	4
Pierdomenico	1998	Italy	NA	NA	42	C: 46 ± 6 N: 47 ± 6	C: 148 ± 11/96 ± 4 N: 122 ± 4/76 ± 4	Case-control	M ± SD	/	7
Sakai	1998	Japan	919	1266	2185	≥40	F: 128.3 ± 20.8/75.8 ± 11.4 M: 134.0 ± 20.0/81.0 ± 11.7	Cross-sectional	Correlation coefficient	Age, sex, BMI, smoking, physical activity	5
Bates	1998	UK	NA	NA	541	≥65	152.1 ± 23.2/78.2 ± 13.1	Cross-sectional	Regression correlation	/	4
Tang	1998	China	NA	NA	84	30-60	NA	Case-control	Correlation coefficient	/	6
Sherman	2000	USA	31	21	52	C: 49 ± 12 N: 5 ± 11	NA	Case-control	M ± SD	/	8
Langlois	2001	Belgium	96	123	219	69 ± 9	C: 147 ± 15/91 ± 12 N: 126 ± 10/75 ± 8	Case-control	M ± SD	/	7
Chen	2002	USA	NA	NA	15317	≥20	C: 144.8 ± 0.49/81.4 ± 0.31 N: 115.6 ± 0.18/71.9 ± 0.18	Cross-sectional	M ± SE OR	Age, sex, race, education, alcohol, BMI	7
Chen	2004	China	28	72	100	19-40	NA	Cross-sectional	Correlation coefficient	/	4
Rodrigo	2007	Chile	66	/	66	35-60	C: 137.5 ± 0.2/91.9 ± 1.3 N: 119.5 ± 0.8/78.2 ± 0.8	Cross-sectional	M ± SD Correlation coefficient	/	5
Kumar	2014	Malaysia	NA	NA	690	56-64	C: 143.57 ± 11.31/91.87 ± 8.77 N: 119.71 ± 5.67/76.78 ± 4.62	Case-control	M ± SD	/	8
Zhou	2014	China	28	72	100	19-40	NA	Cross-sectional	Correlation coefficient	/	5
Naregal	2017	India	NA	NA	60	60-80	C: 146.03 ± 11.37/75.33 ± 5.21 N: 113.76 ± 3.18/71.70 ± 5.17	Cross-sectional	M ± SD Correlation coefficient	/	5

C: case, N: non-case, F: female, M: male, M: mean, SD: standard deviation, SE: standard error, OR: odds ratio.

**Table 2:** The outcomes of subgroup analysis and adjustment of confounders.

Subgroups		SBP					DBP				
		Studies (*n*)	Fisher's *Z*	95% CI	*I* ^2^ (%)	*P*_h_	Studies (*n*)	Fisher's *Z*	95% CI	*I* ^2^ (%)	*P*_h_
All studies		11	-0.17	(-0.20, -0.15)	37	0.10	10	-0.15	(-0.20, -0.10)	51	0.02

Sex	Male	5	-0.20	(-0.28, -0.12)	60	0.03	6	-0.15	(-0.24, -0.06)	65	0.01
	Female	2	-0.13	(-0.17, -0.09)	0	0.63	2	-0.12	(-0.20, -0.04)	73	0.05

Hypertension	Yes	2	-0.16	(-0.36,0.03)	86	0.008	2	-0.23	(-0.42, -0.03)	57	0.13
	No	6	-0.24	(-0.32, -0.17)	0	0.54	5	-0.20	(-0.28, -0.13)	11	0.34

Antihypertensive drugs	Unclear	4	-0.15	(-0.18, -0.12)	18	0.30	4	-0.10	(-0.14, -0.06)	0	0.69
	No	7	-0.22	(-0.27, -0.16)	14	0.32	7	-0.17	(-0.23, -0.12)	35	0.16

Level of vitamin A and vitamin E	Normal	2	-0.53	(-0.79, -0.28)	0	0.69	2	-0.26	(-0.51, -0.00)	59	0.12
	Abnormal	3	-0.19	(-0.25, -0.13)	45	0.16	3	-0.18	(-0.24, -0.12)	57	0.10

Area	America	3	-0.31	(-0.42, -0.20)	55	0.08	3	-0.23	(-0.34, -0.11)	0	0.45
	Europe	2	-0.17	(-0.21, -0.13)	0	0.67	2	-0.16	(-0.20, -0.12)	0	0.38
	Asia	6	-0.17	(-0.20, -0.13)	22	0.26	5	-0.13	(-0.20, -0.05)	61	0.02

Sample size	<100	4	-0.37	(-0.50, -0.25)	32	0.21	3	-0.16	(-0.30, -0.02)	53	0.09
	100-500	4	-0.21	(-0.28, -0.14)	0	0.99	4	-0.21	(-0.28, -0.14)	20	0.29
	>500	3	-0.16	(-0.19, -0.13)	4	0.37	3	-0.13	(-0.16, -0.10)	54	0.09

Adjusted Confounders											
Age	Male	2	-0.12	(-0.17, -0.07)	75	0.04	2	-0.12	(-0.16, -0.07)	51	0.15
	Female	2	-0.10	(-0.14, -0.06)	14	0.28	2	-0.09	(-0.14, -0.05)	46	0.17
Age+sex		2	-0.11	(-0.14, -0.08)	0	0.65	2	-0.11	(-0.14, -0.08)	0	0.85
Age+BMI	Male	2	-0.11	(-0.16, -0.07)	78	0.03	2	-0.10	(-0.15, -0.06)	67	0.08
	Female	2	-0.09	(-0.13, -0.04)	0	0.52	2	-0.09	(-0.13, -0.05)	6	0.30
Age+sex+BMI		2	-0.09	(-0.12, -0.06)	0	0.50	2	-0.10	(-0.13, -0.07)	0	0.72

*P*_h_: *P* value of heterogeneity.

## Data Availability

All data generated or analyzed during this study are included in this article.

## Abbreviations

CVDs: Cardiovascular diseases
BP: Blood pressure
PubMed: National Library of Medicine
WOS: Web of Science
CNKI: China National Knowledge Infrastructure
MOOSE: Systematic Reviews of Observational Studies
SD: Standard deviations
NOS: Newcastle-Ottawa Scale
AHRQ: Agency for Healthcare Research and Quality
SE: Standard error
MD: Mean difference
CI: Confidence intervals
RevMan: Reviewer Manager
SBP: Systolic blood pressure
DBP: Diastolic blood pressure
BMI: Body mass index
AA: Ascorbic acid
GSH: Glutathione
GSH-Px: Glutathione peroxidase.
